# Regioselective alkali metal reduction of dibenzocyclooctadiene lignan derivatives, demethoxylation followed by dehalogenation

**DOI:** 10.1186/s13065-017-0368-z

**Published:** 2017-12-27

**Authors:** Qing-yao Wang, Jia-qi Fang, Lu-lu Deng, Xiao-jiang Hao, Shu-zhen Mu

**Affiliations:** 1 State Key Laboratory of Functions and Applications of Medicinal Plants, Guizhou Medical University, 3491 Baijin Road, Guiyang, 550014 China; 2 The Key Laboratory of Chemistry for Natural Products of Guizhou Province and Chinese Academy of Sciences, 3491 Baijin Road, Guiyang, 550014 China

**Keywords:** Alkali metal, Regioselective demethoxylation, Dehalogenation, Halogenated dibenzocyclooctadiene lignans, Aryl halides

## Abstract

The regioselective demethoxylation and dehalogenation of dihalogenated dibenzocyclooctadiene lignans derivatives were realized in a one-step reaction with excellent yields in the sodium and* t*-butanol reaction system.

## Introduction

Natural dibenzocyclooctadiene lignans are found widely in the Schisandraceae family of flowering plants, and most members of these lignans exhibit a variety of significant biological activities and pharmacological functions [1]. Some of these lignans or their derivatives have become important sources of lead compounds in drug discovery. Indeed, two notable liver protectants, biphenyldicarboxylate (DDB) and bicyclol, have been developed from these natural products and have subsequently been widely used in clinics. In past decades, most structural modifications to dibenzocyclooctadiene lignans have been mainly focused on aromatic protons or the hydroxyl group of the biphenyl ring, including the halogenation or nitration at C-4 and C-11, oxidation at C-8, and esterification or etherification of the hydroxyl group at C-14 [1]. The removal of methoxy groups from the diphenyl skeleton may greatly increase the sites available for chemical modification, which would enable more dibenzocyclooctadiene lignan derivatives to be prepared for drug screening.

Aryl halides are very useful in organic syntheses, such as acting as substrates in transition-metal-catalysed coupling reactions and for the preparation of Grignard reagents. However, their toxicities are the focus in environmental protection measures [2]. Thus far, four different aryl-halide dehalogenation methods involving transition-metal catalysts [2–17], photochemistry [18–21], free-radical reductions [22], and two-electron transfer by super-electron donors have been investigated [22]. Although high yields can be achieved using these methods [10–23], several factors must be considered, including the high cost of expensive metal catalysts, the inaccessibility of organic super-electron donors, strict reaction conditions that always involve high temperatures, long reaction time, and complicated combinations of reagents.

Herein, we establish a novel method that is both facile and uses mild conditions for both the dehalogenation and regioselective demethoxylation of dihalogenated dibenzocyclooctadiene lignans.

**Fig. 1:**
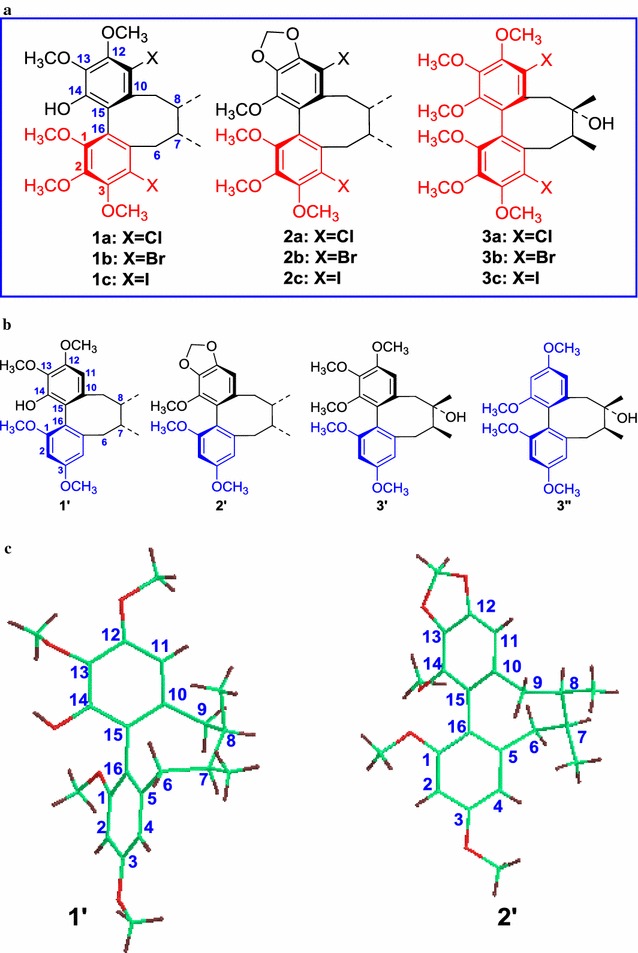
**a** Structures of compounds **1a**–**1c**, **2a**–**2c** and **3a**–**3c**; **b** molecular structures of compounds **1**′–**3**′ and **3**″ prepared in Na/t-BuOH; **c** the single-crystal X-ray structures compounds **1**′-**2**′

In our previous study, we investigated the regioselective demethoxylation of dibenzocyclooctadiene lignans at C-2 and C-13. The reported reaction system involving alkali metals in alcohol [24, 25], THF [26], heptane, and others [27] can be used to regioselectively remove the methoxy group, which is twisted out of the plane of the aromatic system. In this case, the proposed reaction mechanism involves a single-electron transfer, and electron-withdrawing substituents on the aromatic ring have been observed to promote the reaction [27]. However, this method has yet to be used on compounds with the biphenyl ring. In the present study, we used this method to regioselectively remove the methoxy group at C-2 or C-13 of nine dibenzocyclooctadiene lignan halides: halogenated schisandhenol derivatives (**1a**‒**1c**), halogenated schizandrin B derivatives (**2a**‒**2c**), and halogenated schizandrin derivatives (**3a**‒**3c**), as shown in Fig. 1. As expected, three target compounds (i.e., **1**′‒**3**′) were successfully synthesised, as shown in Fig. 1; their structures were confirmed by various techniques, including single-crystal X-ray diffraction (Fig. 1), NMR spectroscopy, and HR-MS.

## Experimental section

### Gerneral

Unless otherwise noted, all solvents and reagents were freshly distilled or purified according to standard procedures. Nuclear magnetic resonance (NMR) spectra were obtained on INOVA-400 MHz NMR spectrometer instrument in the solvent CDCl_3_ at room temperature. High-Resolution Electron Impact Mass Spectra (HR-EI-MS) were performed on Waters Autospec Premier P776 spectrometer. Analytical thin layer chromatography (TLC) was carried out on precoated plates (silica gel GF254), and spots were visualized with ultraviolet (UV) light and 5% H_2_SO_4_ in ethanol. Schisanhenol, schizandrin B and schizandrin were isolated and purified from *Schisandra chinensis*. All reactions were carried out under nitrogen. All reagents were commercially obtained and, where appropriate, purified prior to use. All conversions reported were determined using analytical HPLC with UV detection at 254 nm.

### Synthesis of compound **1**′

To a solution of Schisanhenol (200 mg, 0.498 mmol) in *t*-butanol (10 mL) was added sodium metal (100 eq), the mixture was stirred vigorously under nitrogen at 50 °C until sodium metal dissolved completely. Then water (40 mL) was added and 10% HCl solution was used to acidify the mixture, which was extracted three times with dichloromethane (3 × 50 mL). The combined organic phases were dried over anhydrous Na_2_SO_4_ and evaporated to dryness under reduced pressure. The residue was purified by flash chromatography on silica gel (petroleum ether: ethyl acetate = 7:1) to afford the compound **1**′ as a colorless oil (153 mg, yield 83%), which was further recrystallized from petroleum ether:ethyl acetate = 3:1 to give a colorless rhombic crystal.

Compound **1**′: a colorless rhombic crystal; mp: 156–158 °C; 153 mg, yield 83%; ^1^H-NMR (CDCl_3_, 400 MHz) *δ* (ppm): 6.44 (d, *J* = 2.2 Hz, 1H), 6.41 (d, *J* = 2.2 Hz, 1H), 6.39 (s, 1H), 3.91 (s, 3H), 3.88 (s, 3H), 3.85 (s, 3H), 3.74 (s, 3H), 2.61 (m, 1H), 2.51 (d, *J* = 13.1 Hz, 1H), 2.34 (m, 1H), 2.09 (d, *J* = 13.1 Hz, 1H), 1.91 (m, 1H), 1.83 (m, 1H), 1.02 (d, *J* = 7.2 Hz, 3H), 0.77 (d, *J* = 7.2 Hz, 3H); ^13^C-NMR (CDCl_3_, 100 MHz) *δ* (ppm): 160.3 (C), 158.0 (C), 150.4 (C), 147.0 (C), 146.4 (C), 134.3 (C), 133.6 (C), 116.7 (C), 116.1 (C), 107.1 (CH), 105.4 (CH), 96.2 (CH), 60.9 (OCH_3_), 56.0 (OCH_3_), 55.7 (OCH_3_), 55.2 (OCH_3_), 40.7 (CH), 39.3 (CH_2_), 35.9 (CH_2_), 33.7 (CH), 21.8 (CH_3_), 12.7 (CH_3_); HREIMS *m/z* 372.1939 [M]^+^ (calcd for C_22_H_28_O_5_, 372.1937).

#### X-Ray crystallographic data for compound **1′**

Colorless rhombic crystals of **1′** (petroleum ether-EtOAc) belong to the orthorhombic space group P21 21 21 (19). The crystal data: C_22_H_28_O_5_, M = 372.19, a = 10.6599(4) Å, b = 11.4746(4) Å, c = 16.4505(6) Å, a/b = 0.9290, b/c = 0.6975, c/a = 1.5432, V = 2012.19(12) Å^3^, Z = 4. The crystal structure was solved and refined by the direct method Shelxs-97, expanded using difference Fourier techniques and full-matrix least-squares calculations. Crystallographic data for the structure of **1**′ have been deposited in the Cambridge Crystallographic Data Centre (deposition No. CCDC1037348). These data can be obtained free of charge via http://www.ccdc.com.ac.uk/conts/retrieving.html (or 12 Union Road, Cambridge CB21EZ, UK, Fax: (+44)1223-336-033, e-mail: deposit@ccdc.cam.ac.uk).

### Synthesis of compound **1a**′

To a solution of 4, 11-dichloroschisandhenol (250 mg, 0.531 mmol) in *t*-butanol (10 mL) was added appropriate sodium metal, the mixture was stirred vigorously under nitrogen at 60 °C until sodium metal dissolved completely. Then water (40 mL) was added and 10% HCl solution was used to acidify the mixture, which was extracted three times with dichloromethane (3 × 50 mL). The combined organic phases were dried over anhydrous Na_2_SO_4_ and evaporated to dryness under reduced pressure. The residue was purified by pressure reducing column chromatography on silica gel H (petroleum ether: ethyl acetate = 10: 1) to afford the compounds **1a**′ as a white solid (17 mg, yield 7.0%).

Compound **1a**′: a white solid; 17 mg, yield 7%; ^1^H-NMR (CDCl_3_, 400 MHz) δ (ppm): 6.59 (s, 1H), 5.62 (s, 1H), 3.98 (s, 3H), 3.94 (s, 3H), 3.91 (s, 3H), 3.87 (s, 3H), 3.61 (s, 3H), 2.39 (m, 2H), 2.12 (m, 1H), 1.99(m, 1H), 1.78 (m, 1H), 1.01 (d, *J* = 7.6 Hz, 3H), 0.81 (d, *J* = 6.8 Hz, 3H); ^13^C-NMR (CDCl_3_, 100 MHz) *δ* (ppm): 153.5 (C), 151.1 (C), 147.9 (C), 145.7 (C), 139.7 (C), 139.6 (C), 138.4 (C), 133.1 (C), 120.5 (C), 120.4 (C), 120.4 (C), 107.6 (CH), 61.2 (OCH_3_), 61.0 (OCH_3_), 60.9 (OCH_3_), 60.7 (OCH_3_), 55.8 (OCH_3_), 41.3 (CH), 35.7 (CH_2_), 34.5 (CH), 34.4 (CH_2_), 21.6 (CH_3_), 10.2(CH_3_). HREIMS m/z 436.1653 [M]^+^ (calcd for C_23_H_29_O_6_Cl, 436.1653).

### Synthesis of compound **1b**′

To a solution of 4, 11-dibromoschisandhenol (250 mg, 0.447 mmol) in *t*-butanol (10 mL) was added appropriate sodium metal, the mixture was stirred vigorously under nitrogen at 60 °C until sodium metal dissolved completely. Then water (40 mL) was added and 10% HCl solution was used to acidify the mixture, which was extracted three times with dichloromethane (3 × 50 mL). The combined organic phases were dried over anhydrous Na_2_SO_4_ and evaporated to dryness under reduced pressure. The residue was purified by pressure reducing column chromatography on silica gel H (petroleum ether: ethyl acetate = 10: 1) to afford the compounds **1b**′ as a yellowish solid (21 mg, yield 9.8%).

Compound **1b**′: a yellowish solid; 21 mg, yield 9.8%; ^1^H-NMR (CDCl_3_, 400 MHz) *δ* (ppm): 6.58 (s, 1H), 5.66 (s, 1H), 3.97 (s, 3H), 3.92 (s, 6H), 3.90 (s, 3H), 3.60 (s, 3H), 2.40 (m, 2H), 2.14 (d, *J* = 13.2 Hz, 1H), 1.96 (m, 1H), 1.76 (m, 2H), 1.00 (d, *J* = 7.2 Hz, 3H), 0.84 (d, *J* = 7.2 Hz, 3H); ^13^C-NMR (CDCl_3_, 100 MHz) *δ* (ppm) 153.4 (C), 151.0 (C), 148.7 (C), 146.3 (C), 139.6 (C), 139.4 (C), 138.3 (C), 134.6 (C), 120.6 (C), 120.6 (C), 112.1 (C), 107.4 (CH), 61.1 (OCH_3_), 61.0 (OCH_3_), 60.8 (OCH_3_), 60.5 (OCH_3_), 55.8 (OCH_3_), 41.2 (CH), 37.0 (CH_2_), 35.8 (CH_2_), 34.4 (CH), 21.2 (CH_3_), 10.6 (CH_3_). HREIMS *m/z* 480.1141 [M]^+^ (calcd for C_23_H_29_O_6_Br, 480.1148).

### Synthesis of compound **2**′

To a solution of 4, 11-dihalogenoschizandrin B (200 mg, 0.5 mmol) in *t*-butanol (10 mL) was added sodium metal (100 eq), the mixture was stirred vigorously under nitrogen at 50 °C until sodium metal dissolved completely. Then water (40 mL) was added and 10% HCl solution was used to acidify the mixture, which was extracted three times with dichloromethane (3 × 50 mL). The combined organic phases were dried over anhydrous Na_2_SO_4_ and evaporated to dryness under reduced pressure. The residue was purified by Semi-HPLC (acetonitrile:methanol:water = 65:5:30) to afford the compound **2**′ (157 mg, yield 85%), which was further recrystallized from petroleum ether: ethyl acetate = 3:1 to give a colorless rhombic crystal.

Compound **2**′: a colorless rhombic crystal; mp: 153–155 °C; 157 mg; yield 85%; ^1^H-NMR (CDCl_3_, 400 MHz) *δ* (ppm): 6.50 (s, 1H), 6.41 (s, 2H), 5.95 (d, *J* = 2.0 Hz, 1H), 5.92 (d, *J* = 2.0 Hz, 1H), 3.84 (s, 3H), 3.80 (s, 3H), 3.73 (s, 3H), 2.63 (m, 1H), 2.54 (m, 1H), 2.27 (m, 1H), 2.03 (m, 1H), 1.92 (m, 1H), 1.79 (m, 1H), 0.97 (d, *J* = 9.0 Hz, 3H), 0.75 (d, *J* = 9.0 Hz, 3H); ^13^C-NMR (CDCl_3_, 100 MHz) *δ* (ppm); 158.6 (C), 158.2 (C), 148.4 (C), 141.1 (C), 140.5 (C), 137.9 (C), 134.6 (C), 121.2 (C), 118.3 (C), 108.3 (CH), 103.3 (CH), 100.7 (CH_2_), 96.0 (CH), 59.5 (OCH_3_), 55.7 (OCH_3_), 55.2 (OCH_3_), 40.7 (CH), 39.2 (CH_2_), 35.5 (CH_2_), 33.5 (CH), 21.6 (CH_3_), 12.6 (CH_3_); HREIMS *m/z* 370.1779 [M]^+^ (calcd for C_22_H_26_O_5_, 370.1780).

#### X-ray crystallographic data for compound **2**′

Colorless rhombic crystals of **2**′ (petroleum ether-EtOAc) belong to the triclinic space group P – 1(2). The crystal data: C_22_H_26_O_5_, M = 370.17, a = 10.3652(8) Å, b = 12.4132(10) Å, c = 16.0181(12) Å, α = 107.486(7)°, β = 90.929(6)°, γ = 104.611(7)°, V = 1892.69(30)  Å^3^, Z = 2. Crystallographic data for the structure of **2**′ have been deposited in the Cambridge Crystallographic Data Centre (deposition No. CCDC1037304). These data can be obtained free of charge via http://www.ccdc.com.ac.uk/conts/retrieving.html (or 12 Union Road, Cambridge CB21EZ, UK, Fax: (+44)1223-336-033, e-mail: deposit@ccdc.cam.ac.uk).

### Synthesis of compounds **3**′ and **3**′′

To a solution of the schisandrin (200 mg, 0. 463 mmol) in *t*-butanol (10 mL) was added sodium metal (100 eq), the mixture was stirred vigorously under nitrogen at 50 °C until sodium metal dissolved completely. Then water (40 mL) was added and 10% HCl solution was used to acidify the mixture, which was extracted three times with dichloromethane (3 × 50 mL). The combined organic phases were dried over anhydrous Na_2_SO_4_ and evaporated to dryness under reduced pressure. The residue was purified by pressure reducing column chromatography on silica gel H (petroleum ether:ethyl acetate = 10:1) to afford the compounds **3**′ and **3**′′ as the faint yellow oil (33 mg, yield 18%; 112 mg, yield 65%).

Compound **3**′: a faint yellow oil; 33 mg; yield 18%; ^1^H-NMR (CDCl_3_, 400 MHz) *δ* (ppm); 6.48 (s, 1H), 6.40 (d, *J* = 2.4 Hz, 1H), 6.39 (d *J* = 2.4 Hz, 1H), 3.81(s, 3H), 3.80 (s, 3H), 3.79 (s, 3H), 3.65 (s, 3H), 3.53 (s, 3H), 2.64 (m, 2H), 2.34 (m, 2H), 1.81 (m, 1H), 1.19 (s, 3H), 0.77 (d, *J* = 7.2 Hz, 3H); ^13^C-NMR (CDCl_3_, 100 MHz) *δ* (ppm); 159.5 (C), 158.6 (C), 151.7 (C), 151.7 (C), 140.0 (C), 138.2 (C), 134.0 (C), 122.5 (C), 119.3 (C), 110.4 (CH), 107.3 (CH), 97.4 (CH), 71.8 (C), 60.8 (CH_3_), 60.7 (CH_3_), 55.8 (2 × CH_3_), 55.3 (CH_3_), 41.7 (CH), 41.1 (CH_2_), 34.5 (CH_2_), 29.8 (CH_3_), 15.8 (CH_3_); HREIMS *m/z* 402.2046 [M]^+^ (calcd for C_23_H_30_O_6_, 402.2042).

Compound **3**″: a faint yellow oil; 112 mg; yield 65%; ^1^H-NMR (CDCl_3_, 400 MHz) *δ* (ppm); 6.46 (m, 4H), 3.85 (s, 3H), 3.84 (s, 3H), 3.70 (s, 6H), 2.73 (m, 2H), 2.42 (m, 2H), 1.91 (m, 1H), 1.26 (d, *J* = 9.5 Hz, 3H), 0.85 (d, *J* = 9.5 Hz, 3H); ^13^C-NMR (CDCl_3_, 100 MHz) *δ* (ppm); 159.3 (C), 158.9, (C) 158.7 (C), 158.5 (C), 140.3 (C), 138.2 (C), 117.8 (C), 115.2 (C), 108.4 (CH), 107.4 (CH), 97.6 (CH), 96.5 (CH), 71.8 (C), 55.9 (CH_3_), 55.8 (CH_3_), 55.2 (CH_3_), 55.1 (CH_3_), 41.7 (CH), 41.0 (CH_2_), 34.5 (CH_2_), 29.9 (CH_3_), 15.7 (CH_3_); HREIMS *m/z* 372.1942 [M]^+^ (calcd for C_22_H_28_O_5_, 372.1937).

## Results and discussion

In our study, 4, 11-dichlororschisandhenol (**1a**) was designed and synthesised. Surprisingly, the demethoxylation of the dichloro analogue could easily occur in an alkali-alcohol reaction system; in addition, two chlorine atoms of **1a** were also removed, as shown in Table 1.

**Table 1:** Optimization for reaction conditions of dehalogenation and regioselective demethoxylation of 4, 11-dichloroschisandhenol (**1a**)
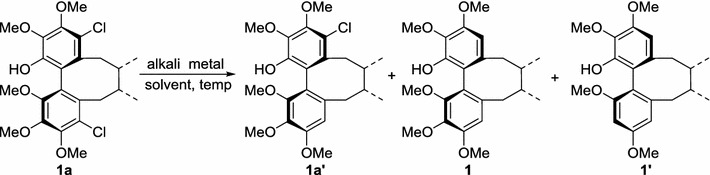

Entry^a^	Alkalimetal	Eq.^b^	Solvent	[C] (mM)	Temp (°C)	Time (h)	Products (conv^c^, %)
1	Na	100	Absolute ethanol	14	rt	2	**1a**′ (0); **1** (0); **1**′ (47)
2	Na	100	*t*-Butanol	9	rt	24	**1a**′ (0); **1** (0); **1**′ (63)
3	K	100	*t*-Butanol	14	50	3	**1a**′ (0); **1** (0); **1**′ (40)
4	K	30	Dry THF	5	rt	24	**1a**′ (0); **1** (1); **1**′ (41)
5	Na	100	*t*-Butanol	5	60	< 4	**1a**′ (7); **1** (8); **1**′ (56)
6	Na	100	*t*-Butanol	5	40	12	**1a**′ (0); **1** (29); **1**′ (0)
7	Na	100	*t*-Butanol	5	50	4	**1a**′ (0); **1** (1); **1**′ (83)
8	Na	50	*t*-Butanol	5	50	< 4	**1a**′ (1); **1** (2); **1**′ (48)
9	Na	75	*t*-Butanol	5	50	< 4	**1a**′ (0); **1** (3); **1**′ (61)
10	Na	125	*t*-Butanol	5	50	12	**1a**′ (1); **1** (2); **1**′ (86)
11	Na	125	*t*-Butanol	4	50	5	**1a**′ (0); **1** (0); **1**′ (53)

^a^For all reactions, 20 mg (0.0426 mmol) substrate was used

^b^Equivalent of alkalimetal

^c^Conversion of the product was determined by HPLC with UV detection at 254 nm

According to the literature [24, 25, 28], the regiospecific demethoxylation of aromatic rings could be achieved with a higher yield in a reaction system composed of Na/*t*-butanol or potassium metal in dry THF. Thus, we designed and performed various experiments aimed at optimising the reaction conditions by investigating the dehalogenation and regioselective demethoxylation of 4, 11-dichloroschisandhenol (**1a**), as shown in Table 1.

A comparison of entries 1 and 2 in Table 1 reveals that dehalogenation and regioselective demethoxylation using sodium metal in *t*-butanol provided a higher yield than that recently reported for sodium metal in absolute ethanol [24, 28]. However, potassium metal was not better than sodium metal, irrespective of whether the reaction was performed in dry THF or *t*-BuOH (Table 1, entries 2–4), as evidenced by the longer reaction time and lower yields. More importantly, some byproducts were produced. Reactions performed at 50 °C required only a moderate reaction time. In these cases, the highest yields were obtained (Table 1, entries 5–7). When the optimal equivalent amounts of sodium metal were examined (Table 1, entries 7–10), more equivalents of sodium were observed to increase the product yield. However, the highest equivalent of sodium (125 eq, with a total conversion of 90%) required quite long reaction time to dissolve completely. Moreover, significant improvement in product yield compared with 100 eq of sodium metal (total conversion of 85%) was not attained. In addition, the molarity of the substrate was observed to affect the product yield (Table 1, entries 10 and 11), and 5 mmol/L of the substrate was observed to be the optimal molarity in this case. Given all of the aforementioned factors, when the reaction was completed until all the sodium metal was dissolved, the optimised reaction conditions were established as: 5 mmol/L as the molarity of the substrate,* t*-butanol as the reaction solvent, 100 eq sodium metal as the catalyst, and 50 °C as the reaction temperature.

Under these reaction conditions, the dehalogenation and regiospecific demethoxylation of nine dihalogenated analogues (**1a**–**1c**, **2a**–**2c**, and **3a**–**3c**) were performed, as shown in Table 2.

**Table 2:** Dehalogenation and regioselective demethoxylation of **1a**–**1c**, **2a**–**2c** and **3a**–**3c** 
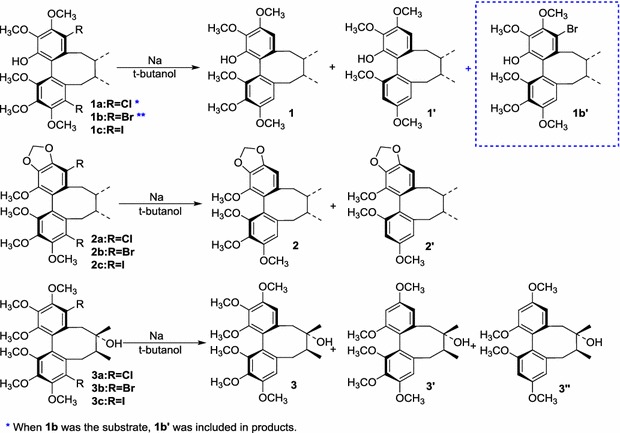

Entry^a^	Substrate	Products (Conv^b^, %)		
1	**1a**	–	**1** (2)	**1**′ (83)
2	**1b**	**1b**′ (5)	**1** (10)	**1**′ (71)
3	**1c**	–	**1** (35)	**1**′ (53)
4	**2a**	–	**2** (16)	**2**′ (62)
5	**2b**	–	**2** (26)	**2**′ (57)
6	**2c**	–	**2** (37)	**2**′ (57)
7	**3a**	**3** (20)	**3**′ (12)	**3**′′ (50)
8	**3b**	**3** (23)	**3**′ (18)	**3**′′ (58)
9	**3c**	**3** (24)	**3**′ (14)	**3**′ (62)

^a^Reaction was performed with substrate (0.0426 mmol), Na (100 eq), *t*-butanol (8 mL) at 50 °C until all of sodium was dissolved

^b^Conversions of the products were determined by HPLC with UV detection at 254 nm

Nine dihalogenated analogues were synthesised and used as substrates in the reaction. Notably, all nine substrates reacted as expected in good yield under the optimised reaction conditions, as shown in Table 2. The detection of the reaction products showed that the dehalogenation occurred prior to the regioselective demethoxylation. The reason for this behaviour may be that the halogen atom has a stronger electron-withdrawing inductive effect and a weaker electron-donating conjugation than the methoxy group. Furthermore, the halogen anion may impart greater stability to the analogues than the methoxy anion. Different dihalogenated analogues exhibited different reactivities, consistent with the bond dissociation energies of aryl halides (Ar–I < Ar–Br < Ar–Cl) [22, 23]. The removal of the iodine atom was easiest, whereas the chlorine atom exhibited the lowest reactivity. Careful observations of the products generated from substrates **1a** and **1b** under the optimised reaction conditions led to the detection of products **1a**′ and **1b**′, which may further confirm that dehalogenation occurred prior to regioselective demethoxylation. These products may have formed because the free hydroxyl group at C-14 reacted with sodium alcoholate in the Na/*t*-butanol reaction system to become a negatively charged oxygen ion; this stronger electron-donating group would greatly increase the electron density of the benzene ring to which it bonded. As a result, this electron-rich benzene ring is unlikely to accept an additional electron from sodium metal as easily as a relatively electron-deficient benzene ring. Thus, the substrates **1a** and **1b** with electron-donating groups exhibited weaker reactivity.

**Scheme 1:**
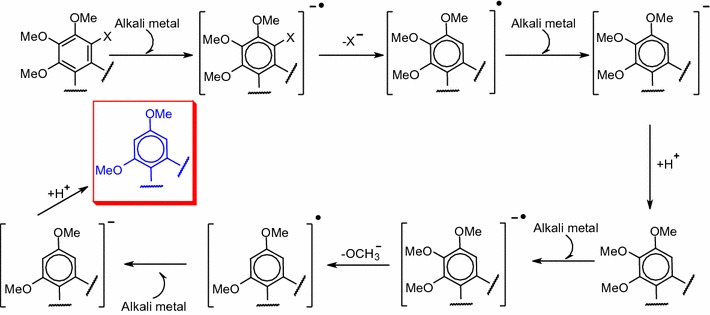
Probable mechanism of the dehalogenation and demethoxylation of dihalogenated dibenzocyclooctadiene lignans derivatives

If the aforementioned results are considered in combination with those reported in the literature [26, 28–32], a probable two-electron transfer mechanism can be proposed (Scheme 1). The alkali metal acts as an electron donor to provide one electron to the substrate. Cleavage of the aryl halide then occurs to form a halogen anion and an aromatic radical. This aromatic radical would prefer another electron from the alkali metal to afford an aromatic anion rather than trigger a cycle of difficult radical reactions because the aromatic radical is unlikely to abstract a hydrogen atom from* t*-butanol [33]. Such a two-electron transfer cycle could occur again to remove an additional methoxy group. In the last step, the terminal aromatic anion may abstract a proton from *t*-butanol to give the final product.

**Scheme 2:**
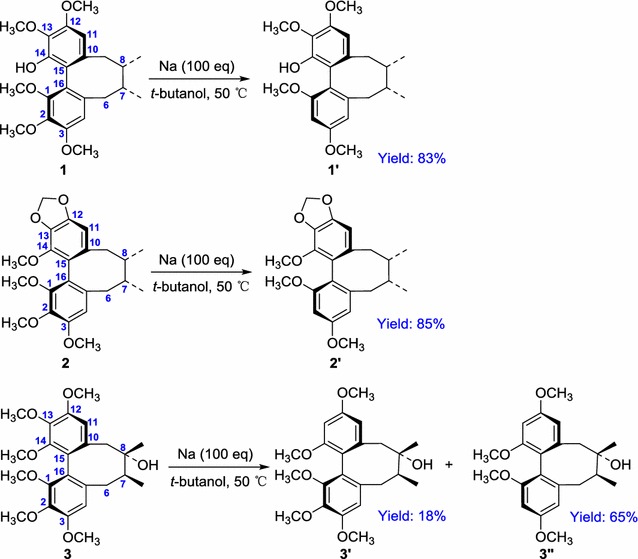
Regioselective demethoxylation of compounds **1**‒**3**

To confirm the above mechanism by further experiments, the demethoxylation of three dibenzocyclooctadiene lignans (**1**‒**3**) was designed and dealt with the above optimised reaction condition at the Na/*t*-butanol reaction system as shown Scheme 2. And just as we predicted, four corresponding demethoxy products (**1**′, **2**′, **3**′ and **3**′′) were obtained with the yield of 83, 85, 18 and 65%, respectively, which revealed that the cleavage of the halogen atoms might occur before that of methoxy group. So the probable two-electron transfer mechanism proposed should be reasonable.

## Conclusions

In conclusion, we have reported a Na/*t*-butanol reaction system that was used for the dehalogenation and regioselective demethoxylation of biphenyl-ring derivatives without forming any byproduct. This simple and mild method that uses an inexpensive catalyst may represent a novel key method for the regioselective demethoxylation of dibenzocyclooctadiene lignans and may serve as an alternative to other methods for the dehalogenation of aryl halides.
